# Feeding soy protein concentrates with low or high isoflavone decreases liver inflammation by reducing lipopolysaccharide translocation

**DOI:** 10.3389/fnut.2023.1278158

**Published:** 2023-11-20

**Authors:** Wei Li, Reza Hakkak

**Affiliations:** ^1^Department of Dietetics and Nutrition, University of Arkansas for Medical Sciences, Little Rock, AR, United States; ^2^Arkansas Children’s Research Institute, Little Rock, AR, United States; ^3^Department of Pediatrics, University of Arkansas for Medical Sciences, Little Rock, AR, United States

**Keywords:** obesity, Zucker rats, liver, LPS translocation, inflammation, soy protein concentrate, isoflavone

## Abstract

Lipopolysaccharide (LPS) translocation and inflammation contribute to the increased risk of chronic diseases, including non-alcoholic fatty liver disease (NAFLD), associated with obesity. Previously, we reported that feeding soy protein with high or low (negligible) isoflavone reduces liver steatosis in obese Zucker rats, and the reduced steatosis is accompanied by decreased serum C-reactive protein levels. The current study investigated the effect of feeding soy protein concentrate (SPC) with high or low isoflavone (HIF or LIF) on liver inflammation and LPS translocation in obese Zucker rats. Six-week-old male lean (L, *n* = 21) and obese (O, *n* = 21) Zucker rats were fed casein control, SPC-LIF, or SPC-HIF diets for 18 weeks. At the end of 18 weeks, the expression levels of tumor necrosis factor-α (TNF-α), monocyte chemoattractant protein-1 (MCP-1), inducible nitric oxide synthase (iNOS), arginase 1 (ARG1), lipopolysaccharide binding protein (LBP), myeloperoxidase (MPO), and sterol regulatory element-binding protein 1 (SREBP-1) were significantly higher in obese rats compared to lean rats. Compared to the casein control diet, both the SPC-LIF and SPC-HIF diets significantly decreased TNF-α, MCP-1, iNOS, and LBP expression in obese rats, which is accompanied by significantly less LPS staining in liver slides from SPC-LIF-and SPC-HIF-fed obese rats compared to the casein control diet-fed obese rats. Taken together, the SPC-LIF and SPC-HIF diets attenuated liver inflammation in obese Zucker rats, likely by decreasing LPS translocation.

## 1 Introduction

Low-grade inflammation contributes to obesity-associated chronic diseases such as cardiovascular disease, type 2 diabetes, certain types of cancer, and non-alcoholic fatty liver disease (NAFLD). In obesity, increased pro-inflammatory cytokine and chemokine secretion from adipose tissue affects organs/tissues such as the liver and skeletal muscle, leading to insulin resistance, ectopic fat deposition, and a wide range of metabolic disturbances (1–5). Traditionally, inflammatory signals that arise from the lipid-laden adipose tissue were considered the main source of systemic inflammation. However, this cannot fully explain the observation that diseases such as NAFLD are associated with increased inflammation after adjusting for confounding variables such as obesity and metabolic syndrome (6, 7). In recent years, aided by a better understanding of the role of gut microbiota in health and diseases, there has been a paradigm shift regarding the origin(s) of obesity-associated inflammation that brings the gastrointestinal (GI) tract into the spotlight (8, 9). GI tract commensal bacteria-derived components, particularly LPS, traverse the portal vein to encounter the liver and enter systemic circulation or are transported by lipoproteins, collectively known as LPS translocation (10). Obesity is associated with altered gut microbiota and increased intestinal permeability, both of which may contribute to increased LPS translocation and subsequent systemic inflammation (11). Elevated blood LPS levels are associated with obesity in humans and animals (12).

LPS translocation is particularly relevant to liver inflammation and liver diseases because the liver is exposed to LPS that traverses the portal vein (13). For example, patients with diagnosed NAFLD have a higher LPS concentration in their circulation compared to healthy controls (14). Levels of LPS in blood and liver biopsy samples also correlate with severity of the disease in NAFLD patients (15). Because NAFLD has no symptoms in most cases and diagnoses rely on imaging or histology, human studies on the relationship between LPS translocation and NAFLD are limited by relatively small sample sizes. The observational nature of human studies reveals only correlations rather than causality. On the other hand, a causal relationship between LPS translocation and NAFLD has been revealed by animal studies. In obese (*fa/fa*) Zucker rats, intraperitoneal LPS injection exacerbates hepatic steatosis (16). Low-dose subcutaneous LPS injection also worsens high-fat diet-induced NAFLD in C57BL/6 mice (17). LPS leads to liver inflammation by triggering macrophages to release inflammatory cytokines, including TNF-α (18, 19). The contribution of TNF-α to the development of NAFLD is supported by the resistance to liver steatosis in TNF-α−/− mice and mice treated with a TNF-α receptor antagonist (20, 21).

In our previous study, we reported that feeding obese Zucker rats SPC-LIF and SPC-HIF diets reduces liver steatosis compared to a casein control diet (22, 23). The reduced hepatic steatosis in obese rats is accompanied by decreased systemic inflammation [i.e., serum C-reactive protein (CRP)] (24). This study was designed to investigate whether there are differences in liver inflammation between lean and obese rats fed casein control, SPC-LIF, and SPC-HIF diets, and, if so, whether different levels of LPS translocation may be a contributing factor. We hypothesized that the SPC-LIF and SPC-HIF diets would reduce liver inflammation and LPS translocation in obese Zucker rats compared to the casein control diet.

## 2 Materials and methods

### 2.1 Ethics statement

The University of Arkansas for Medical Sciences/Arkansas Children’s Research Institute Institutional Animal Care and Use Committee approved the animal care protocol and procedures used in the study. We adhered to the institutional regulations (Protocol code no. 3968; approved on December 20, 2019) and the guidelines of the United States Department of Agriculture (USDA, Washington, DC, United States) Animal Welfare Act.

### 2.2 Experimental design

Six-week-old male lean and obese *(fa/fa)* Zucker rats were purchased from Charles River Laboratories (Wilmington, MA, United States). All rats were acclimated to the AIN-93G rodent diet for a week before the start of the experiment. Lean and obese rats were randomly assigned to one of the three dietary groups with 7 rats to each group and fed a semi-purified diet similar to the AIN-93G diet with dietary protein supplied in the form of casein (control), soy protein concentrate with low isoflavone (SPC-LIF) (Arcon SJ; ADM; Decatur, IL), or soy protein concentrate with high isoflavone (SPC-HIF) (Arcon SM; ADM; Decatur, IL). Rats had *ad libitum* access to water and diets during the experiment. The SPC-LIF diet contained 0.154 mg isoflavone/g protein with an aglycone component of approximately 0.16 mg/g protein (genistein, 0.15 mg/g protein; daidzein, 0.011 mg/g protein; and glycitin, below level of detection). The SPC-HIF diet had 2.153 mg isoflavone/g protein with an aglycone component of approximately 1.72 mg/g protein (genistein, 0.382 mg/g protein; daidzein, 0.216 mg/g protein; glycitin, 0.005 mg/g protein). The casein-based diet does not have a detectable level of isoflavones. L-cystine was added at 3 g/kg to the casein diet and 1.2 g/kg to the SPC-LIF and SPC-HIF diets. L-methionine was added at 2.2 g/kg to the SPC-LIF and SPC-HIF diets to match the L-methionine level in the casein diet. All three diets were made to be isocaloric and isonitrogenous, with detailed compositions reported previously (23). Three identical dietary groups (control, SPC-LIF, and SPC-HIF) were created in lean rats and in obese rats. During week 18, rats were anesthetized with carbon dioxide and euthanized by decapitation. Liver samples were snap frozen in liquid nitrogen and stored at −80°C for subsequent RNA extraction. Two 3-mm sections of each liver lobe were fixed in tissue cassettes and stored in 10% buffered formalin until histological examination and immunohistochemistry.

### 2.3 Quantitation of liver mRNA expression

Total RNA was isolated from frozen liver samples using TRIzol Reagent (15596026, ThermoFisher Scientific, Waltham, MA) according to the protocol provided by the manufacturer. cDNA was synthesized using SuperScript™ First-Strand Synthesis System for RT-PCR (11904018, ThermoFisher Scientific, Waltham, MA). qPCR was performed using the validated TaqMan assays (ThermoFisher Scientific, Waltham, MA) (Table 1) on a QuantStudio 6 Real-time PCR system (Applied Biosystems, Foster City, CA). 18 s rRNA was used as endogenous control, and relative gene expression was calculated using the delta delta Ct (ΔΔCt) method.

**Table 1:** Quantitative PCR assay list.

Gene Name	Species	Assay ID (Thermo Fisher Scientific)
Tnfa	Rat	Rn01525859_g1
Ccl2/Mcp1	Rat	Rn00580555_m1
Il1b	Rat	Rn00580432_m1
Il10	Rat	Rn01483988_g1
iNos/Nos2	Rat	Rn00561646_m1
Arg1	Rat	Rn00691090_m1
Lbp	Rat	Rn00567985_m1
Mpo	Rat	Rn01460205_m1
Srebp1c	Rat	Rn01495769_m1
18 s rRna	Rat	Rn03928990_g1

### 2.4 Immunohistochemistry and image analysis

Formalin-fixed, paraffin-embedded liver samples were microtome-sectioned. Slides were pretreated with Dako Target Retrieval High pH (pH 9.0) buffer (S236784-2, Agilent Technologies, Santa Clara, CA). Liver sections were stained with a primary antibody against LPS (ab35654, Abcam, Cambridge, MA) or a primary antibody against the pan-macrophage marker CD68 (ab283654, Abcam, Cambridge, MA). Biotinylated secondary antibodies, Vector ABC standard Elite HRP Kit (PK-6100, Vector Laboratories, Newark, CA), and Dako DAB (GV82511-2, Agilent Technologies, Santa Clara, CA) were used to visualize antigen staining. Richard-Allan Hematoxylin was used for counterstaining. Slides were scanned at 40× magnification using Aperio Scanscope CS2 (Leica Biosystems, Nußloch, Germany) and subsequently analyzed by Aperio Imagescope software using total pixel count and cytoplasmic algorithms (Leica Biosystems, Nußloch, Germany).

### 2.5 Statistical methods

Quantitative PCR data were analyzed by a two-way ANOVA on obesity status, diet, and the interactions between obesity status and diet, followed by the Fisher’s LSD *post-hoc* tests. Statistical significance was determined at the level of *p* of <0.05. Immunohistochemistry data were analyzed by a one-way ANOVA on diet. Statistical significance was determined at the level of *p* of <0.05. Analyses were performed in SPSS Statistics for Windows, version 28.0.0.0 (IBM Corp., Armonk, NY, United States).

## 3 Results

### 3.1 The effect of soy protein on liver gene expression

**Figure 1:**
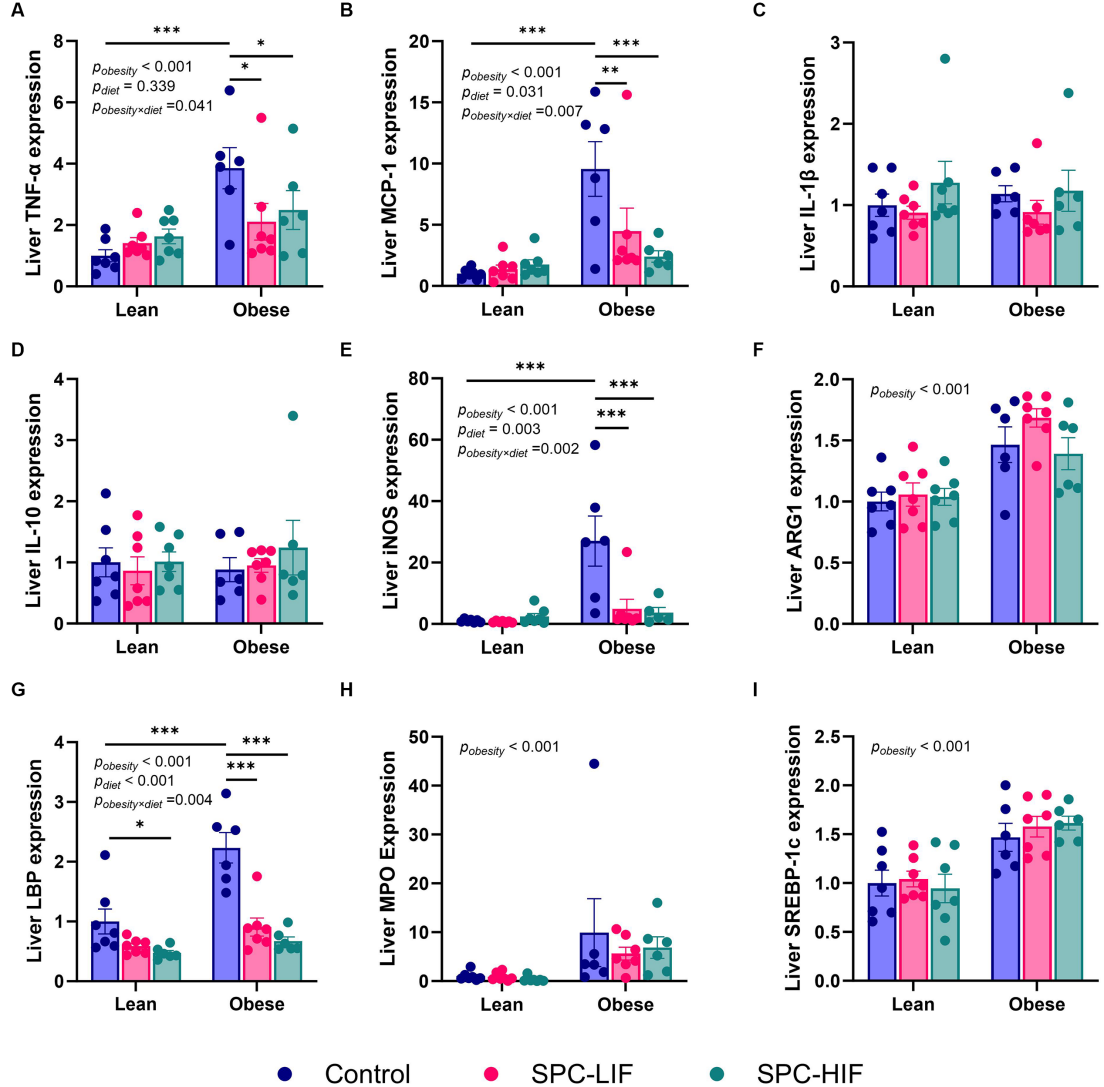
Hepatic TNF-α **(A)**, MCP-1 **(B)**, IL-1β **(C)**, IL-10 **(D)**, iNOS **(E)**, ARG1 **(F)**, LBP **(G)**, MPO **(H)**, and SREBP-1c **(I)** expression measured by TaqMan qPCR. The data are expressed as a ratio to the lean control group. *p < 0.05, **p < 0.01, ***p < 0.001, SPC-LIF, soy protein concentrate with low isoflavone; SPC-HIF, soy protein concentrate with high isoflavone.

After lean and obese Zucker rats were fed a casein control diet, SPC-LIF diet, or SPC-HIF diet for 18 weeks, liver TNF-α, MCP-1, iNOS, and LBP expression levels had significant interactions between diet and obesity status. The expression of TNF-α, MCP-1, iNOS, and LBP was significantly higher in casein control diet-fed obese rats compared to casein control diet-fed lean rats. In obese rats, both the SPC-HIF and SPC-LIF diets significantly decreased TNF-α, MCP-1, iNOS, and LBP expression compared to the casein control diet. In lean rats, the SPC-HIF diet significantly decreased LBP expression compared to the casein control diet (Figures 1A,B,E,G). Liver IL-1β and IL-10 expression did not differ between obese and lean rats or between any dietary groups in obese or lean rats (Figure 1D). There is an effect of obesity status but not diet on liver ARG-1, MPO, and SREBP-1 expression. Obese rats had significantly increased expression of ARG-1, MPO, and SREBP-1 compared to lean rats (Figures 1F,H,I).

### 3.2 The effect of soy protein on liver LPS level In obese rats

**Figure 2:**
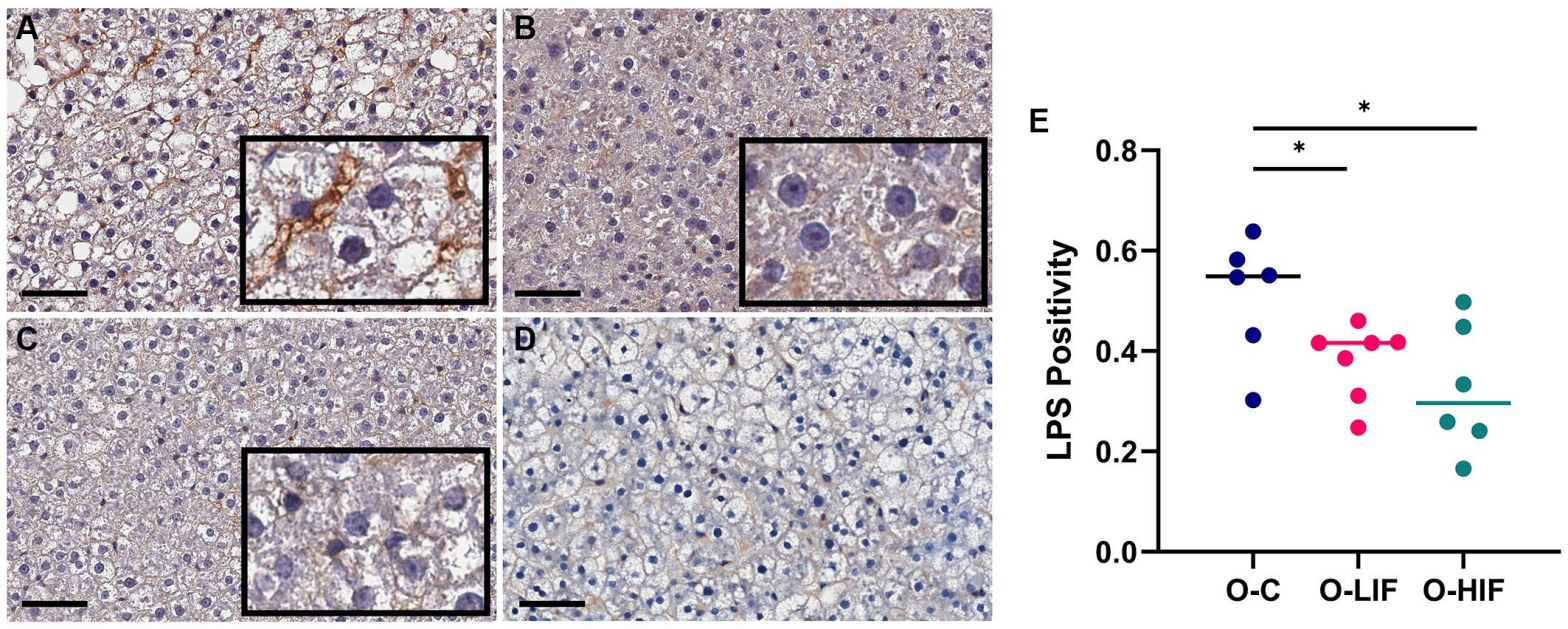
Immunohistochemistry staining for LPS in liver samples from obese (*fa/fa*) Zucker rats. Rats were fed **(A)** a casein control diet, **(B)** a soy protein diet with low isoflavone, and **(C)** a soy protein diet with high isoflavone. **(D)** Negative control for immunohistochemistry: a liver sample stained without primary anti-LPS antibody. **(E)**, LPS positivity (NPositive/NTotal) in all slides was analyzed by a positive pixel count (PPC) algorithm in all samples using Aperio ImageScope software (Leica biosystems). *p < 0.05, O, obese; C, control; LIF, low isoflavone; HIF, high isoflavone.

**Figure 3:**
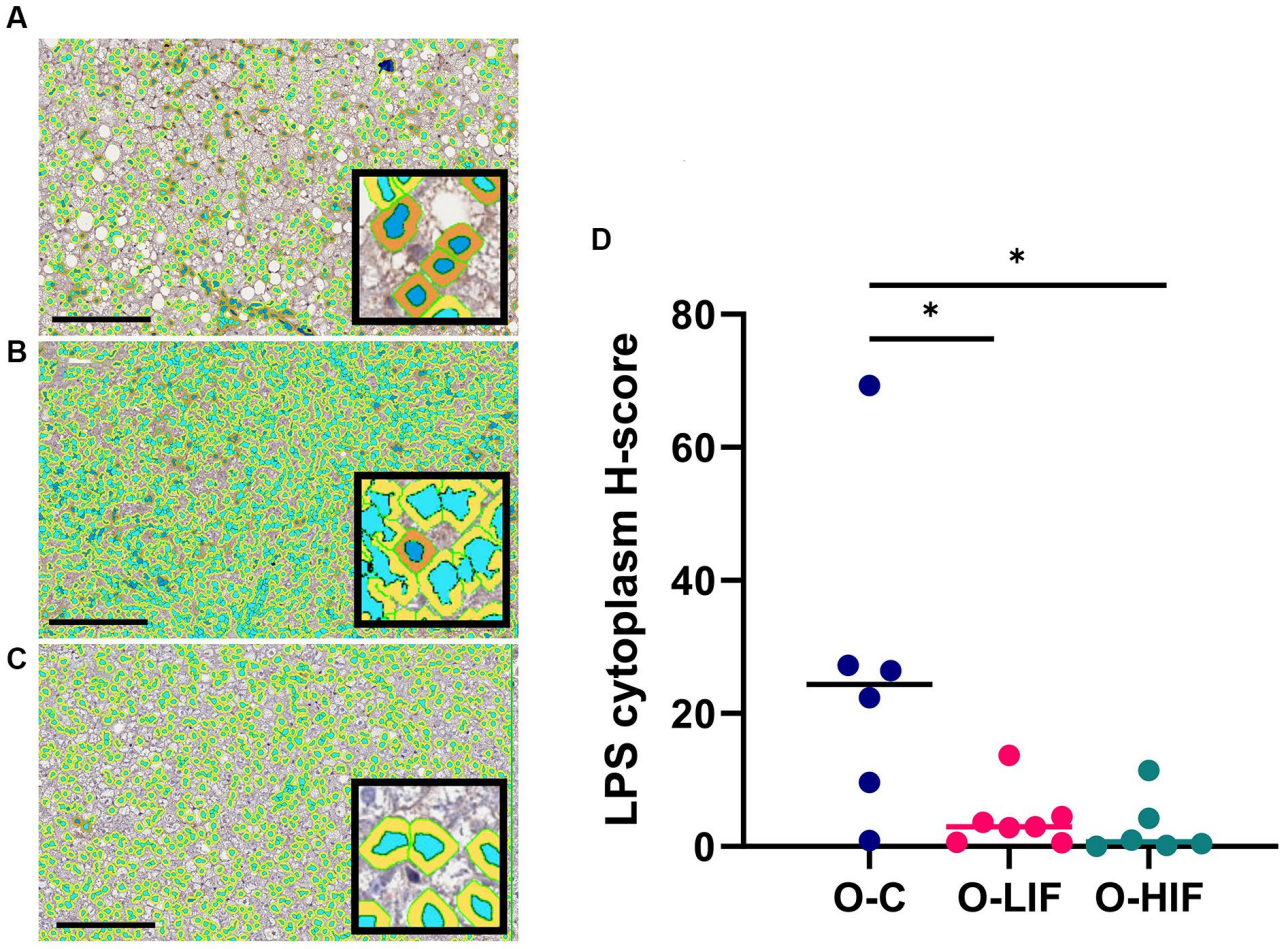
Immunohistochemistry staining for LPS in liver samples from obese (*fa/fa*) Zucker rats analyzed by a cytoplasm algorithm (Aperio ImageScope software, Leica Biosystems). Rats were fed **(A)** a casein control diet, **(B)** a soy protein diet with low isoflavone, and **(C)** a soy protein diet with high isoflavone. The pseudo-blue color defines nuclear areas, and darker cytoplasmic areas indicate greater LPS staining. **(D)** LPS cytoplasm H-score from all samples. *p < 0.05. O, obese; C, control; LIF, low isoflavone; HIF, high isoflavone.

Because the liver expression levels of pro-inflammatory genes TNF-α, MCP-1, iNOS, and LBP were decreased by feeding SPC-LIF and SPC-HIF diets in obese rats, we analyzed liver samples from obese rats fed the casein control diet, the SPC-LIF diet, and the SPC-HIF diet for LPS translocation by immunohistochemistry. Representative images from the casein control group (Figure 2A), the SPC-LIF group (Figure 2B), and the SPC-HIF group (Figure 2C) all showed positive LPS staining, while a negative control sample had minimum to zero staining (Figure 2D). When the immunohistochemistry images were quantified by a positive pixel count algorithm (Aperio ImageScope, Leica) and compared between groups, liver sample LPS positivity was significantly lower in the SPC-LIF and SPC-HIF diet-fed obese rats compared to the casein control diet-fed obese rats (Figure 2E). Because the LPS-induced inflammatory response requires endocytosis of the LPS TLR4 complex (25), we quantified cytoplasmic LPS in the immunohistochemistry images using a cytoplasmic algorithm (Aperio ImageScope, Leica). In the pseudo-color-coded representative images from a casein control diet-fed obese rat (Figure 3A), a SPC-LIF diet-fed obese rat (Figure 3B), and a SPC-HIF diet-fed obese rat (Figure 3C), both SPC-LIF and SPC-HIF samples appeared to have a lower frequency of cells staining strongly positive for cytoplasmic LPS (orange pseudo-color). When data from all slides were analyzed, LPS cytoplasm H-scores were significantly lower in SPC-LIF and SPC-HIF diet-fed obese rats compared to casein control diet-fed obese rats (Figure 3D).

### 3.3 The effect of soy protein on liver macrophage marker CD68

**Figure 4:**
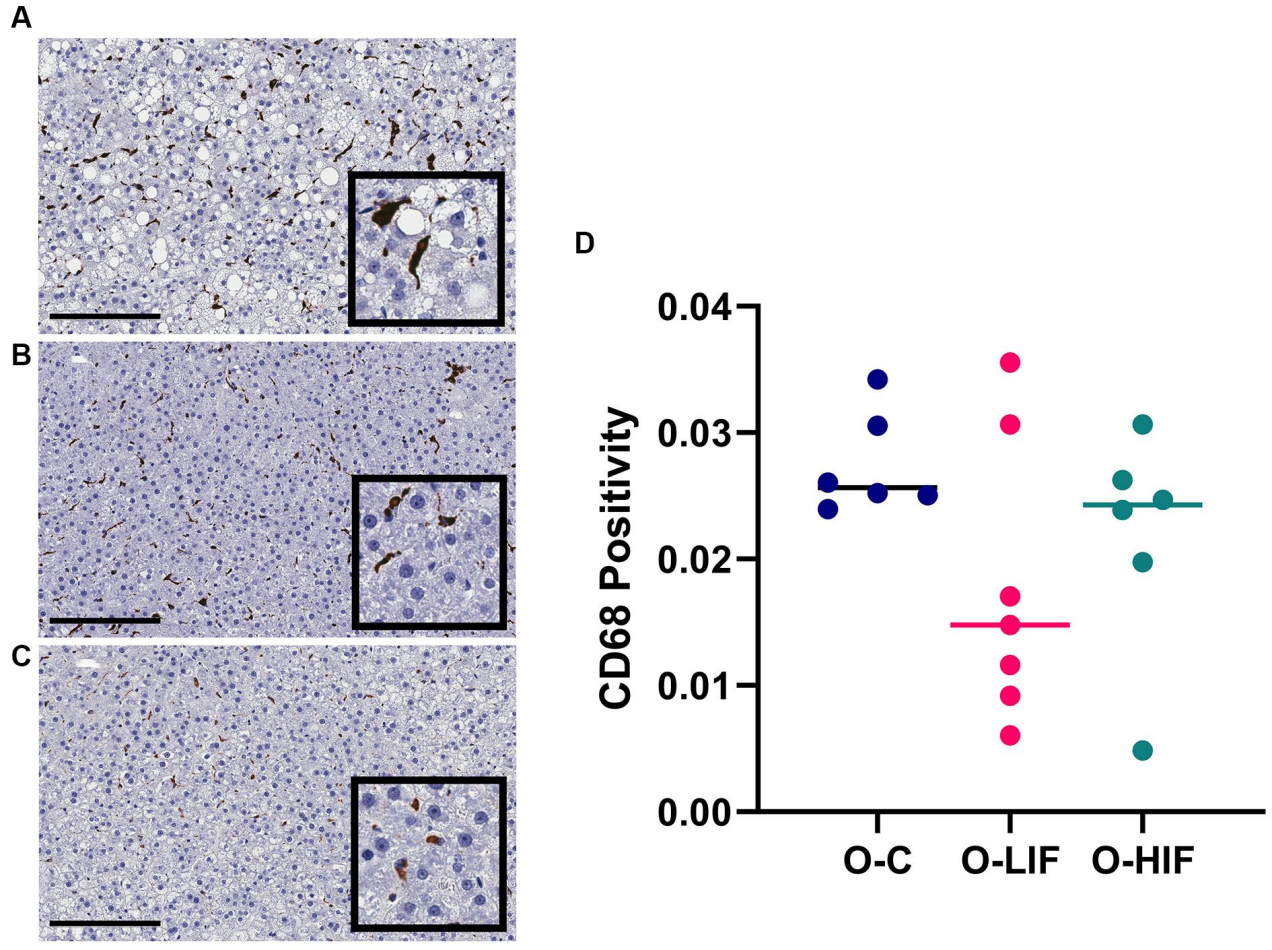
Immunohistochemistry staining for CD68 in liver samples from obese (fa/fa) Zucker rats. Rats were fed **(A)** a casein control diet, **(B)** a soy protein diet with low isoflavone, and **(C)** a soy protein diet with high isoflavone. **(D)** CD68 positivity (NPositive/NTotal) analyzed by a positive pixel count (PPC) algorithm in all samples using Aperio ImageScope software (Leica biosystems). O, obese; C, control; LIF, low isoflavone; HIF, high isoflavone.

When obese rat liver samples were stained for the pan-macrophage marker CD68 by immunohistochemistry, samples from the casein control group (Figure 4A), the SPC-LIF group (Figure 4B), and the SPC-HIF group (Figure 4C) had positive CD68 staining. When the immunohistochemistry images were quantified by a positive pixel count algorithm (Aperio ImageScope, Leica), there were no significant differences in CD68 positivity between any groups (Figure 4D).

## 4 Discussion

Soy foods have anti-inflammatory properties that may mediate their health benefits by reducing the risk of chronic diseases (26–28). In previous studies, we reported that feeding obese Zucker rats SPC-LIF and SPC-HIF diets protected the liver against steatosis and reduced systemic inflammation compared to a casein control diet (23, 24). In the current study, we investigated whether feeding obese Zucker rats SPC-LIF and SPC-HIF diets would reduce liver inflammation and LPS translocation. We found that obese rats had increased expression of a number of pro-inflammatory genes, including TNF-α, MCP-1, and iNOS, in the liver compared to lean rats, while their expression levels were decreased in obese rats by SPC-LIF and SPC-HIF diets. As a master pre-inflammatory cytokine, TNF-α is primarily produced by activated macrophages (29). The contribution of TNF-α to the development of NAFLD is supported by the resistance to liver steatosis in TNF-α^−/−^ mice and mice treated with a TNF-α receptor antagonist (20, 21). MCP-1 is a chemokine produced primarily by activated macrophages to recruit monocytes to the site of inflammation (30). iNOS is a hallmark marker of M1 (pro-inflammatory, classically activated) macrophages (31). The increased TNF-α, MCP-1, and iNOS expression in the liver of obese rats compared to lean rats and their decreased expression in the SPC-LIF and SPC-HIF diet-fed obese rats compared to the casein control diet-fed obese rats suggest that liver pro-inflammatory macrophage activity was increased by obesity, and the increase was attenuated by soy protein diets.

We did not detect any effect of obesity or diet on liver IL-10 or IL-1β expression. IL-10 is an anti-inflammatory cytokines produced by T cells, B cells, and macrophages (32). Our data indicate that IL-10 is not involved in the development of liver steatosis or the effect of soy protein on liver steatosis in obese Zucker rats. Although IL-1β, a macrophage-derived pro-inflammatory cytokine, did not differ between obese and lean rats or between any dietary groups, we speculate that it may be because the increased inflammatory stimuli in obesity were not sufficient to increase IL-1β expression.

Because it has been reported by others that neutrophils may contribute to the development of NAFLD (33), we also measured the expression of the neutrophil marker MPO in the liver samples. Obesity increased liver MPO expression, but there was no effect of any diet (Figure 1H), suggesting that the liver neutrophil activity was increased by obesity but changes in neutrophil activity did not mediate the protective effect of soy protein on liver steatosis. There was also an increase in ARG1 expression in obese rats. Initially, we chose to measure ARG1 as a marker for anti-inflammatory M2 type macrophages, and we expected that soy protein diets may increase ARG1 in obese rats compared to the casein control diet. However, ARG1 is a urea cycle enzyme that is abundant in hepatocytes. Its increased expression in the obese groups was most likely not an indication of M2 macrophage activity but rather an upregulation of urea cycle enzymes due to the increased food/protein intake by obese rats compared to lean controls. The high level of ARG1 expression by hepatocytes may have masked any potential difference in ARG1 expression in macrophages. Finally, SREBP-1c expression levels were also higher in obese rats but not affected by diets. The mammalian genome encodes three SREBP isoforms, namely, SREBP-1a, SREBP-2, and SREBP-1c. All SREBPs are activators of lipid synthesis, with SREBP-1c being the predominate form in the liver that activates fatty acid synthesis (34). Liver-specific expression of SREBP-1c is associated with fatty liver (35). Our results suggest that obese Zucker rats had increased liver lipogenesis compared to lean rats, but SREBP-1c expression did not mediate the effect of soy protein on liver steatosis.

Liver LBP expression was significantly higher in obese rats compared to lean rats and decreased by the SPC-LIF and SPC-HIF diets to levels similar to those of lean rats (Figure 1G). Because increased LBP is an indicator for LPS translocation (36, 37), we measured LPS levels in the liver of obese rats by immunohistochemistry and showed that the SPC-LIF and SPC-HIF diet-fed rats had significantly less liver LPS staining compared to casein control diet-fed obese rats (Figure 2). Similar to total LPS staining, the cytoplasmic LPS levels were also significantly lower in SPC-LIF and SPC-HIF diet-fed obese rats compared to casein control diet-fed obese rats (Figure 3). The differences in cytoplasmic LPS staining were likely due to differences in LBP expression. In aqueous environments such as plasma and interstitial fluids, LPS molecules form aggregates due to their amphipathic nature. LBP interacts with LPS aggregates and, together with CD14, extracts LPS molecules as monomers to facilitate their interaction with the TLR-4 receptor complex (25, 38). Endocytosis of LPS and the TLR-4 complex initiates a cascade of intracellular signaling events that lead to the activation of the NFκB pathway and increased expression of inflammation cytokines (25). The increased cytoplasmic LPS staining that we observed is consistent with the increased expression of LBP and its subsequent effect on LPS-TLR4 endocytosis.

Finally, we stained the liver slides of obese rats with the pan-macrophage marker CD68 to investigate whether the decrease in inflammation markers by the SPC-LIF and SPC-HIF diets was accompanied by a decreased number of macrophages. The results showed no difference in CD68 positive pixel counts between obese rats fed different diets (Figure 4D), indicating that the numbers of macrophages are similar between groups. However, macrophages in the liver of SPC-LIF and SPC-HIF diet-fed obese rats appeared to aggregate less compared to casein control diet-fed obese rats, as reflected by smaller foci of positive staining (Figures 4A–C).

Although both SPC-LIF and SPC-HIF diet-fed obese rats attenuated liver inflammation and LPS translocation compared to casein control diet-fed obese rats, there were no significant differences between the SPC-LIF and SPC-HIF groups. Soy isoflavones have a wide range of health benefits. The benefits of soy isoflavones are dependent on food sources that affect bioavailability and the interaction of isoflavones with the intestinal microbiota (39). For example, certain bacterial populations can produce equol, a beneficial bacterial metabolite derived from daidzein. There are equol producers and non-producers in the human population (40). Previously, we reported the bioavailability of isoflavones from SPC in lean and obese Zucker rats (24). Rats fed the SPC-HIF diet have significantly higher serum levels of genistein, daidzein, and equol compared to rats fed the SPC-LIF diet (24). When taken together with the lack of additional effects of the SPC-HIF diet compared to the SPC-LIF diet in the current study, we concluded that the anti-inflammatory effect of SPC and the reduction of LPS translocation by SPC in obese Zucker rats could mostly be attributed to the protein rather than the isoflavone components of SPC. The anti-inflammatory effects of some soy protein-derived peptides have been reported. Soy-derived angiotensin-converting enzyme (ACE)-inhibitory peptide inhibits inflammation in vascular smooth muscle cells (41). Soy peptides also protect against LPS-induced inflammation in cultured intestinal cells by reducing nitric oxide and inflammatory cytokine expression and cultured macrophages by suppressing TLR4-mediated pathways such as NFκB activation (42, 43). Soy-derived tripeptide Phe-Leu-Val reduces TNF-α-induced inflammation and insulin resistance in adipocytes (44). Although most of these reports have come from *in vitro* experiments, soy-derived peptide can be absorbed into the circulation from the GI tract (45). For example, the soy peptide lunasin has been shown to have anticancer effects *in vivo* (46). The protective effects of soy bioactive peptides against chronic diseases have been reviewed in detail elsewhere, with many of their benefits attributed to their anti-inflammatory functions (47, 48).

There are two main potential mechanisms for soy protein’s inhibition of LPS translocation in obese rats. First, soy protein may optimize the intestinal microbiota and therefore reduce the origin of LPS from its source. The effects of soy foods on intestinal microbiota composition, in particular the reduction of pathogenic bacterial populations by soy foods, have been reported in both animal and human studies (39). Second, soy protein may promote the barrier function of the intestinal epithelium to reduce its permeability to LPS. The effects of soy protein on intestinal permeability have been investigated in animal models with mixed results. In zebra fish, soybean meal increases intestinal permeability independent of the microbiota (49). In weaned piglets, a high dose of soybean agglutinin (SBA) increases intestinal permeability, while a low dose of SBA exerts no effect (50, 51). In mice, feeding SPC reduces colonic inflammation and prevents the loss of gut barrier function induced by dextran sulfate sodium (DSS) (52). The effects of soy protein on intestinal function and microbiota will form the basis of our future investigations.

## 5 Conclusion

Feeding the SPC-LIF and SPC-HIF diets to obese Zucker rats significantly reduced obesity-induced liver inflammation, likely by decreasing LPS translocation. Based on these results, we will design future studies to investigate the mechanisms of soy protein’s inhibitory effect on LPS translocation. Because increased LPS translocation has been implicated in the development of other obesity-associated chronic diseases, including type 2 diabetes, cancer, and Alzheimer’s disease (53, 54), an in-depth mechanistic understanding of soy protein’s inhibition of LPS translocation during obesity may shed light on soy protein’s wide range of health benefits.

## Data availability statement

The original contributions presented in the study are included in the article/supplementary material, further inquiries can be directed to the corresponding author.

## Ethics statement

The animal study was approved by University of Arkansas for Medical Sciences/Arkansas Children’s Research Institute Institutional Animal Care and Use Committee and adhered to the institutional regulations. The study was conducted in accordance with the local legislation and institutional requirements.

## Author contributions

WL: Formal analysis, Investigation, Methodology, Validation, Writing – original draft, Writing – review & editing, Conceptualization, Data curation, Resources. RH: Formal analysis, Investigation, Methodology, Validation, Writing – original draft, Writing – review & editing, Funding acquisition, Project administration, Supervision, Resources.
